# Self-Emulsifying Drug Delivery System Enhances the Antidiabetic Activity of *Passiflora ligularis* Leaf Extract

**DOI:** 10.3390/pharmaceutics17060730

**Published:** 2025-05-31

**Authors:** Sandra M. Echeverry, Diana P. Rey, Ivonne H. Valderrama, Ingrid A. Rodriguez, Paula M. Sepúlveda, Bibiana Verlindo de Araujo, Fátima Regina Mena Barreto Silva, Diana Marcela Aragón

**Affiliations:** 1 Departamento de Farmacia, Universidad Nacional de Colombia, Av. Carrera 30 # 45-03 Edif. 450, Bogotá 111321, Colombia; smecheverryg@unal.edu.co (S.M.E.); dpreyp@unal.edu.co (D.P.R.); ihvalderramap@unal.edu.co (I.H.V.); inarodriguezma@unal.edu.co (I.A.R.); pmsepulvedar@unal.edu.co (P.M.S.); 2 Programa de Pós-Graduação em Ciências Farmacêuticas, Faculdade de Farmácia, Universidade Federal do Rio Grande Do Sul (UFRGS), Av. Ipiranga, 2752, Porto Alegre 90610-000, RS, Brazil; bibiana.araujo@ufrgs.br; 3 Instituto de Bioeletricidade Celular (IBIOCEL),Ciência & Saúde, Departamento de Bioquímica, Centro de Ciências Biológicas, Universidade Federal de Santa Catarina, Rua João Pio Duarte Silva, 241, Sala G 301, Florianópolis 88038-000, SC, Brazil; mena.barreto@ufsc.br

**Keywords:** *Passiflora*, diabetes, flavonoids-*O*-glucoside, oxidative stress, superoxide dismutase, catalase, malondialdehyde, hypolipidemic

## Abstract

**Background/Objectives:** Previous studies have shown that unformulated extracts of *Passiflora ligularis* leaves exhibit promising antidiabetic activity. This research aimed to demonstrate that formulating the extract into a self-emulsifying drug delivery system (PLE-SEDDS) enhanced its antidiabetic activity in a high-fat-diet/streptozotocin-induced diabetic mouse model. **Methods**: Blood glucose levels (BGLs) of diabetic mice were monitored during 21 days of oral administration of *P. ligularis* extract (PLE) and PLE-SEDDS. Control groups included metformin (positive control), vehicle, and SEDDS vehicle (negative controls). The animals underwent an oral glucose tolerance test (OGTT). The oxidative stress markers superoxide dismutase (SOD), catalase (CAT), and lipid peroxidation quantified by malondialdehyde (MDA) levels were measured in the kidney, liver, and pancreas, complemented with histopathological analysis. Additionally, plasma lipid profile parameters were evaluated. **Results**: The PLE-SEDDS formulation demonstrated superior efficacy compared to the PLE extract in improving antidiabetic outcomes. Animals treated with PLE-SEDDS exhibited a minimal increase in blood glucose levels (11.5%) during the OGTT, compared to 27.4% with PLE and over 77% in the vehicle groups. PLE-SEDDS also showed greater enhancement of SOD and CAT activity, along with a more pronounced reduction in MDA levels, indicating stronger protection against oxidative stress. Histological analysis revealed significant preservation of pancreatic islets, and lipid profile analysis showed greater reductions in triglycerides, cholesterol, and LDL-C, alongside increased HDL-C levels. **Conclusions**: Altogether, these findings suggest that PLE-SEDDS exhibits superior antihyperglycemic, hypolipidemic, and antioxidant effects compared to the unformulated extract, making this novel formulation a promising option for treating type 2 diabetes mellitus.

## 1. Introduction

Diabetes is a complex, chronic endocrine disorder, characterized by elevated blood glucose levels due to insufficient insulin production, or the body’s inability to use insulin effectively. This condition is associated with severe metabolic complications [1]. With diabetes projected to affect approximately 783 million adults by 2045—90% of which are expected to be type 2 diabetes cases [2]—there is a pressing need to explore diverse treatment strategies.

Over the past decades, natural products have emerged as a significant source for anti-diabetic drug discovery. However, the scientific validation of plant-derived drugs remains limited [3]. This limitation arises from challenges such as the need for comprehensive phytochemical, pharmacological, and toxicological analyses, alongside bioavailability studies and pharmacokinetic and pharmacodynamic profiling [3,4]. Furthermore, ensuring consistency and potency in final product formulations is critical [5].

To address these challenges, nanotechnology has gained significant attention for its potential to enhance the delivery and efficacy of herbal extracts in antidiabetic treatments. Among the innovative nanocarriers, self-emulsifying drug delivery systems (SEDDSs) stand out as lipid-based systems composed of oil, surfactant, and cosurfactant, which form fine oil-in-water emulsions upon mild agitation in the gastrointestinal (GI) tract [6]. These systems are versatile and effective for both hydrophilic and lipophilic drugs, including compounds prone to enzymatic degradation [7]. By facilitating the release of active substances directly in their dissolved form, SEDDSs enhance gastrointestinal absorption through passive diffusion, paracellular, fusion, and transcytosis pathways. Additionally, SEDDSs utilize the intestinal lymphatic system to bypass first-pass metabolism, significantly improving bioavailability [8].

Progress in overcoming these barriers has been demonstrated in studies conducted within our research group, investigating the antidiabetic potential of *Passiflora ligularis* leaves. The pharmacological activity of this plant is attributed to *O*-glycosylated flavonoids (mainly derived from the aglycones quercetin, kaempferol, and chrysin). These compounds act through multiple mechanisms, including the activation of MAPK and MEK/ERK pathways, stimulation of GLUT-4 transporter protein synthesis to enhance glucose uptake, facilitation of calcium entry into pancreatic beta-cells, and the inhibition of α-amylase and α-glucosidase enzymes [9,10,11]. The antidiabetic activity of *P. ligularis* has been validated in previous studies, where aqueous and ethanolic extracts demonstrated efficacy in a chronic type 2 diabetes mellitus (T2DM) model [12]. Our ongoing research revealed that the self-emulsifying drug delivery system (PLE-SEDDS) enhances the antihyperglycemic activity of the standardized, unformulated extract (PLE) in an acute in vivo model [13,14]. However, the impact of SEDDSs on other diabetic parameters in a chronic model remains unexplored.

To bridge this gap, the present study aimed to evaluate whether the PLE-SEDDS could enhance the antidiabetic effects of the PLE, in a chronic T2DM model induced by a high-fat diet and streptozotocin in mice.

## 2. Materials and Methods

### 2.1. Materials and Reagents

The optimized *P. ligularis* leaves extract (PLE) was obtained following methodologies described in previous studies of our research group [13,14]. The PLE contents total flavonoids of 38.754 ± 1.48 µg isoquercetin equivalents/mg extract, 5.924 ± 0.196 µg isoquercetin/mg extract, and 2.324 ± 0.112 µg astragalin/mg extract. A voucher specimen was deposited at the Colombian National Herbarium (COL 602878).

The glucose (G7021), metformin, streptozotocin (572201), Lipid Peroxidation (MDA) Assay Kit (MAK085), Catalase Assay Kit (CAT100), and SOD Assay Kit (19160) were obtained from Sigma-Aldrich^®^ (St. Louis, MO, USA). Serum insulin levels were determined using an ELISA kit (Monobind Inc., Lake Forest, CA, USA; Cat. No. 2425-300A)). Lipid markers were analyzed using diagnostic kits from Linear Chemicals S.L.U. (Montgat, Barcelona, Spain), including the Cholesterol MR Kit (Cat. No.1118005), HDL-Cholesterol Kit (Cat. No.1133010), Triglycerides MR Kit, and LDL-Cholesterol Kit (Cat. No.1133105). Castor oil, Cremophor EL (polyethoxylated-35 castor oil), and poly dimethylsiloxane-co-(3-(2-(2-hydroxyethoxy) ethoxy)propyl]methylsiloxane (PDMSHEPMS) were purchased from Sigma-Aldrich^®^ (St. Louis, MO, USA). 

A high-fat diet (Test Diet^®^ DIO Rodent Purified Diet w45% Energy from Fat-Red 58V8) sourced from LabDiet^®^ (St. Louis, MO, USA) was used to induce diabetes.

### 2.2. Preparation and Characterization of the Self-Emulsifying Drug Delivery System Loaded with P. ligularis Extract

The PLE-SEDDS was prepared following the methodology described by Echeverry et al. [14]. The preconcentrate was prepared by dissolving the PLE extract in propylene glycol, followed by vortex mixing and ultrasonic bath treatment using an Elmasonic S 15 H (Elma Schmidbauer GmbH, Singen, Germany). Subsequently, Cremophor^®^ EL, castor oil, and the silicone copolymer PDMSHEPMS (poly dimethylsiloxane-co-(3-(2-(2-hydroxyethoxy)ethoxy)propyl]) were added.

The PLE-SEDDS was characterized for droplet size, polydispersity index (PDI), zeta potential, and distribution coefficient (Log D). For size and PDI, 100 mg of the preconcentrate was diluted in 10 mL of phosphate buffer (50 mM, pH 6.8), gently agitated, and analyzed by dynamic light scattering (Zetasizer Nano ZSP, Malvern Instruments Ltd., Malvern, Worcestershire, UK). Zeta potential was determined using a 1% (*w*/*v*) dilution in deionized water with the same equipment and disposable capillary cells. All measurements were conducted at 37 °C. Log D was assessed to estimate the release behavior of flavonoids. Excess extract was added to phosphate buffer (pH 6.8) and HCl (pH 1.2), stirred at 37 °C for 24 h, then centrifuged (6000 rpm, 15 min) and filtered (0.45 µm PVDF). The quantification of total flavonoids in the PLE extract and PLE-SEDDS was performed using a previously validated high-performance liquid chromatography (HPLC) method, following ICH guidelines. Briefly, chromatographic separation was carried out on a Phenomenex-Luna^®^ C18 column (150 × 4.6 mm, 5 µm; Phenomenex Inc., Torrance, CA, USA) using a gradient elution with water containing 1% formic acid (mobile phase A) and acetonitrile (mobile phase B) at a flow rate of 1.0 mL/min and column temperature of 45 °C. The injection volume was 10 µL, and detection was conducted at 350 nm for total flavonoids, with isoquercetin used as the calibration standard. The method demonstrated excellent linearity in the range of 1.2 to 292.3 µg/mL (r^2^ = 0.999), with limits of detection and quantification of 0.15 and 0.43 µg/mL, respectively. Accuracy and precision were within acceptable limits, with recovery values between 98.4% and 107.7% and intra- and inter-day relative standard deviations below 5% [14].

### 2.3. Animals and Treatment

Type 2 DM was induced according to the methodology described by Rey et al. [12]. Briefly, female CD-1 mice (100–120 days old, weighing 20–25 g) were housed under optimal conditions (22 ± 2 °C, 12 h light/dark cycles; water ad libitum) and randomly divided into two groups: a control group of normoglycemic mice (*n* = 6) that were fed a standard diet (Rodent LabDiet 5001); and mice that were fed a high-fat diet for eight weeks before diabetes mellitus induction.

Diabetes was induced using two intraperitoneal injections of streptozotocin (STZ, 40 mg/kg) administered three days apart. Blood glucose levels (BGLs) were measured using the Accu-Chek Performa^®^ system, with blood collected from the lateral tail vein. Mice with BGLs > 150 mg/dL were selected and randomly assigned to the following treatment groups (with 6 animals each):

1. Vehicle: distilled water;
2. Self-emulsifying drug delivery system (SEDDS) vehicle: formulation without the *P. ligularis* extract;
3. Metformin: 250 mg/kg;
4. *P. ligularis* extract (PLE): 250 mg/kg;
5. *P. ligularis* extract loaded self-emulsifying drug delivery system (PLE-SEDDS): doses 250 mg/kg of *P. ligularis* extract.

During the 21-day treatment period, diabetic mice remained on a high-fat diet, and treatments were administered daily via oral gavage. Meanwhile, the normoglycemic group received saline injections instead of STZ and was orally administered the vehicle to replicate the stress conditions experienced by the diabetic mice.

Weight gain and BGL were recorded weekly. At the end of the treatment period, oral glucose tolerance tests (OGTTs) and insulin resistance index (HOMA-IR) calculations were performed. The animals were sacrificed using intraperitoneal Euthanex^®^ followed by decapitation for blood collection and lipid profile analysis. The blood samples were centrifuged to extract the serum supernatant. Pancreas, liver, and kidney tissues were collected for oxidative stress marker evaluation and histopathological study. The serum and organ samples were stored at −80 °C until use.

### 2.4. Oral Glucose Tolerance Test (OGTT)

The OGTT was performed as per a previously established methodology [15]. Animals were fasted for 4 h before baseline BGLs measurement. Treatments were administered, and 30 min later, a glucose overload (2000 mg/kg) was given via oral gavage. BGLs was measured at various time points using the Accu-Chek Performa^®^ system.

### 2.5. Insulin Resistance Index (HOMA-IR)

Serum insulin levels were measured using an ELISA kit, and insulin resistance (IR) was calculated using the homeostasis model assessment index, as shown in Equation (1) [16]:

(1) $$HOMA-IR=\frac{Plasma\;insulin\;level\;(\mu U/mL)\times Fasting\;plasma\;glucose\;(mg/dL)}{405}$$

### 2.6. Histopathological Analysis

Selected organs were preserved in a 10% phosphate-buffered formaldehyde solution (1:20 ratio), embedded in paraffin, sectioned into 5 µm slices, and stained with hematoxylin and eosin. Histopathological analyses were conducted at the Pathology Laboratory of the Faculty of Medicine, National University of Colombia.

### 2.7. Oxidative Stress Markers and Lipid Profile

Biochemical parameters were assessed using Sigma-Aldrich^®^ commercial kits for catalase (CAT), superoxide dismutase (SOD), and lipid peroxidation, expressed as malondialdehyde (MDA) levels in tissue homogenates.

Diagnostic kits were also used to determine serum lipid profile parameters, including triglycerides, total cholesterol, high-density lipoprotein cholesterol (HDL-C), and low-density lipoprotein cholesterol (LDL-C).

### 2.8. Statistical Analysis

Data were analyzed using GraphPad Prism^®^ software (version 7, San Diego, CA, USA). Results are presented as mean ± SEM (blood glucose levels, oxidative stress parameters) or mean ± SD (serum lipid profile, fasting glucose, fasting insulin, and HOMA index). Statistical significance between treatment groups for oxidative stress markers, lipid profile, and OGTT results was set at *p* ≤ 0.05.

## 3. Results

### 3.1. Self-Emulsifying Drug Delivery System Loaded with P. ligularis Extract

The formulation, previously optimized [14], employed in this study demonstrated favorable physicochemical characteristics for oral delivery. The formulation, composed of castor oil, Cremophor^®^ EL, propylene glycol, and PDMSHEPMS in a 31:120:80:30 ratio with 20% extract loading, yielded nanoemulsions with a mean droplet size of 45.93 ± 1.02 nm, indicating efficient self-emulsification. The polydispersity index (PDI) was 0.27 ± 0.03, reflecting a narrow size distribution suitable for stable formulations. The zeta potential was −10.92 ± 0.42 mV, suggesting moderate physical stability with sufficient repulsive electrostatic interactions to prevent aggregation. Furthermore, the system exhibited favorable partitioning behavior with Log D values of 1.73 ± 0.06 at pH 6.8 and 2.71 ± 0.11 at pH 1.2, indicating moderate affinity toward the lipid phase while ensuring rapid release under gastrointestinal conditions without precipitation risk.

### 3.2. Effect of Treatments on Weight Gain and Blood Glucose Levels

**Figure 1:**
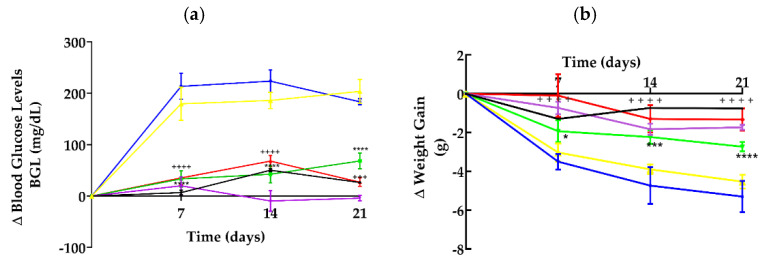
(**a**) Changes in blood glucose levels in diabetic mice. (**b**) Change in weight of diabetic animals. Each group was *n* = 6. Data are expressed as the mean ± SEM. Black: normoglycemics, blue: vehicle, yellow: SEDDS vehicle, violet: metformin 250 mg/kg, green: *P. ligularis* extract 250 mg/kg, red: PLE-SEDDS. Two-way ANOVA post-Bonferroni test; * *p* < 0.05; *** *p* < 0.01; and **** *p* < 0.0001 compared to the vehicle group; ++++ *p* < 0.0001 compared to the SEDDS vehicle.

Figure 1 illustrates the changes (∆) in blood glucose levels (BGLs) and weight gain from day 0 to days 7, 14, and 21 across all treatment groups. The results demonstrate that animals administered with the vehicle (distilled water) and self-emulsifying drug delivery system vehicle increased serum glucose levels by 83.4% and 77.6%, respectively, compared to their baseline values on day 0. In contrast, animals treated with the *P. ligularis* extract (PLE) and *P. ligularis* extract loaded self-emulsifying drug delivery system (PLE-SEDDS) showed substantially lower increases in the BGL, at 27.4% and 11.5%, respectively. Notably, the blood glucose-lowering effect of PLE-SEDDS was comparable to that of metformin (250 mg/kg), which exhibited a 12.8% increase, suggesting a similar glycemic control profile.

Regarding weight changes, animals in both vehicle-treated groups experienced the most significant weight loss, approximately 15%. However, no statistically significant differences were observed between these two groups. In the normoglycemic group, as depicted in Figure 1b, the animals exhibited a non-significant weight loss compared to their initial weight. This lack of weight gain may be attributed to the stress induced by daily oral administration via gavage. In contrast, animals in the PLE and PLE-SEDDS groups demonstrated markedly reduced weight losses of 7.9% and 0.2%, respectively.

### 3.3. Oral Glucose Tolerance Tests

**Figure 2:**
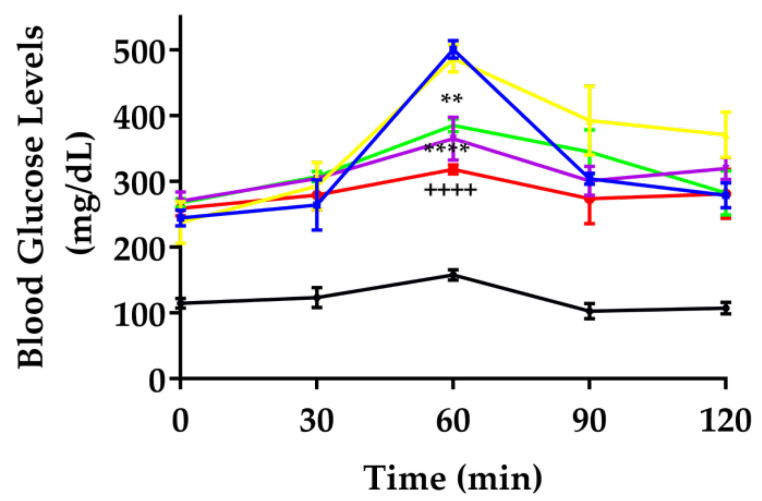
Oral glucose tolerance test. Each group was *n* = 6. Data are expressed as the mean ± SEM. Black: normoglycemics, blue: vehicle, yellow: SEDDS vehicle, violet: metformin 250 mg/kg, green: *P. ligularis* extract 250 mg/kg, red: PLE-SEDDS. Two-way ANOVA post-Bonferroni test; ** *p* < 0.01; and **** *p* < 0.0001 compared to the vehicle group; ++++ *p* < 0.0001 compared to the SEDDS vehicle.

The oral glucose tolerance test (OGTT) in murine models primarily assesses glucose metabolism and insulin sensitivity. The aim is to evaluate how effectively the animal can regulate blood glucose levels following an overload glucose oral test [17,18]. Figure 2 illustrates blood glucose levels in diabetic mice after an oral glucose challenge. At minute 60, corresponding to 30 min after the glucose load, animals in the SEDDS vehicle and vehicle groups exhibited a significant BGL increase of 104%. In contrast, animals treated with the extract and SEDDS formulation showed BGL increases of only 44% and 23%, respectively, suggesting that *P. ligularis* treatments effectively prevent abrupt spikes in BGL following glucose overload. Beyond the 60 min mark, no significant differences in BGL were observed between treatment groups, except for the normoglycemic group.

### 3.4. Insulin Resistance Index (HOMA-IR)

The HOMA-IR index was determined to assess insulin resistance in diabetic rodents. Blood glucose levels (BGLs) and insulin samples were collected after a 4 h fasting period. The HOMA-IR indices for the vehicle and SEDDS vehicle groups were 22.903 ± 0.995 and 24.830 ± 2.422, respectively. In contrast, the extract-treated group displayed a value of 12.164 ± 0.388, and the PLE-SEDDS formulation group exhibited an index of 9.785 ± 0.880. These results indicate that the *P. ligularis* treatments effectively mitigated insulin resistance. Furthermore, the formulation demonstrated a lower HOMA-IR index than metformin (13.610 ± 1.343), although this difference was not statistically significant.

### 3.5. Histological Evaluation of Endocrine and Exocrine Pancreatic Tissue and Other Organs

**Figure 3:**
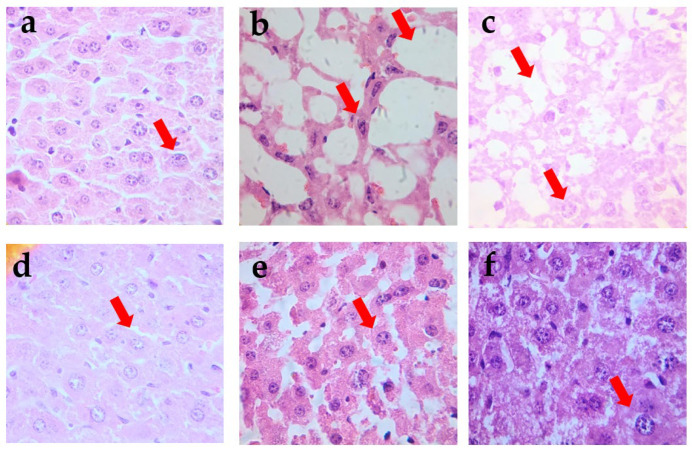
Photomicrographs of pancreatic tissues from different experimental groups were stained with hematoxylin and eosin (H&E) and examined at 40× magnification. (**a**) Normoglycemic group, (**b**) vehicle, (**c**) SEDDS vehicle, (**d**) metformin, (**e**) *P. ligularis* extract, (**f**) PLE-SEDDS formulation. Arrows shows lesion on the exocrine tissue.

Based on the micrographs presented in Figure 3, preserved endocrine tissue, including pancreatic islets, was observed in samples corresponding to the metformin-treated group, the extract, and the PLE-SEDDS formulation. Additionally, the exocrine component displayed normal histological features, with no evidence of necrosis or associated inflammatory infiltrates. In contrast, micrographs B and C, corresponding to the vehicle groups, revealed an absence of endocrine components in the vehicle group. Although a minimal endocrine component was detected in the SEDDS vehicle group, cytoplasmic vacuolar degeneration was observed.

In other evaluated organs, the histological analysis of samples of PLE and PLE-SEDDS treatment groups demonstrated preserved kidney architecture, including intact glomeruli, tubules, and blood vessels with normal histology. Furthermore, perirenal adipose tissue showed no histological alterations in either treatment group.

For the liver, histopathological examination of samples from rodents treated with the standardized extract and PLE-SEDDS formulation revealed preserved hepatic architecture with no signs of necrosis or inflammatory infiltrates. Additionally, no evidence of cholestasis was detected in liver tissue samples from any of the *P. ligularis*-treated groups.

### 3.6. Superoxide Dismutase (SOD) Activity

Significant differences in superoxide dismutase (SOD) activity were observed in liver and kidney tissues compared to the vehicle group. In the liver, SOD activity increased by 78.6% following treatment with the extract, whereas the PLE-SEDDS formulation led to a markedly higher increase of 99.9%. Notably, no significant difference was observed between the PLE-SEDDS and metformin treatments, aligning with histopathological findings that demonstrated preserved liver histology.

**Figure 4:**
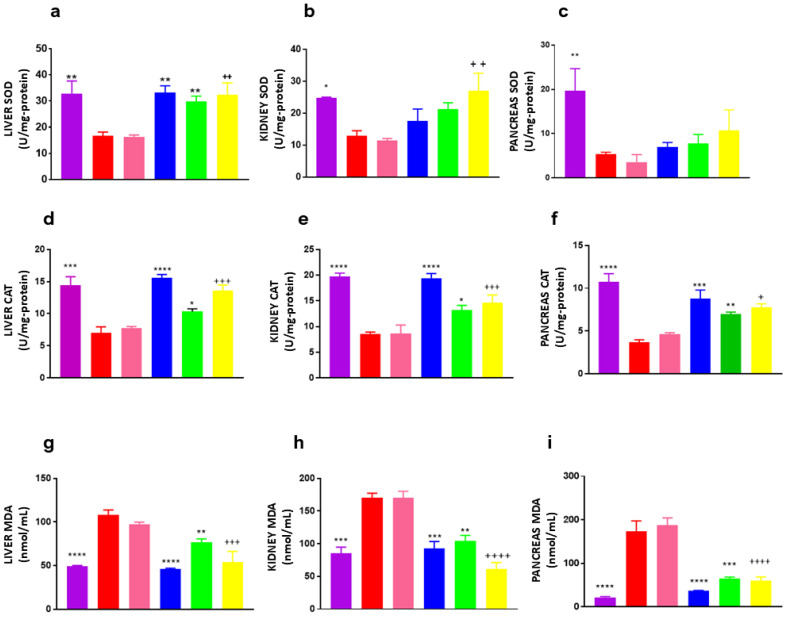
Effect of SEDDS loaded *P. ligularis* on oxidative stress parameters. Violet: normoglycemics, red: vehicle, pink: SEDDS vehicle, blue: metformin 250 mg/kg, green: *P. ligularis* extract 250 mg/kg, yellow: PLE-SEDDS. (Self-emulsifying system loaded *P. ligularis* extract (250 mg/kg)). SOD activity: (**a**) liver, (**b**) kidney, (**c**) pancreas; CAT activity: (**d**) liver, (**e**) kidney, (**f**) pancreas; MDA levels: (**g**) liver, (**h**) kidney, (**i**) pancreas. Data are expressed as mean ± SEM, n = 6 animals per group. One-way ANOVA post-test Dunnet; * *p* < 0.05, ** *p* < 0.01, *** *p* < 0.001 and **** *p* < 0.0001 with respect to the vehicle group and ^+^ *p* < 0.05, ^++^ *p* < 0.01, ^+++^ *p* < 0.001 and ^++++^ *p* < 0.0001 with respect to the SEDDS vehicle group.

In the kidney, the extract enhanced SOD activity by 64.0%, while the PLE-SEDDS formulation exhibited a superior effect, increasing SOD activity by 109.0%. Although SOD activity in the pancreas did not show statistically significant differences, a slight increase was noted (Figure 4a–c). These findings underscore the enhanced antioxidant potential of PLE-SEDDS compared to PLE alone, likely attributable to its improved bioavailability and cellular uptake, further supporting its therapeutic advantage in diabetes management.

### 3.7. Catalase (CAT) Activity

Significant differences in catalase (CAT) activity were observed across all examined tissues (liver, kidney, and pancreas) when comparing the extract and PLE-SEDDS treatments to the vehicle group (Figure 4d–f). Notably, the PLE-SEDDS formulation demonstrated a greater enhancement in CAT activity across all tissues compared to the extract, with the most pronounced effect observed in the liver. Specifically, CAT activity in the liver increased by 94.8% with PLE-SEDDS, whereas the extract treatment led to a lower increase of 48.0%.

In the kidney, CAT activity rose by 69.5% with PLE-SEDDS, compared to 54.6% with the extract. Similarly, in the pancreas, the PLE-SEDDS formulation resulted in a 96.9% increase in CAT activity, outperforming the extract, which induced a 77.9% increase (Figure 4d–f).

### 3.8. Lipid Peroxidation (MDA Levels)

Both the extract and the PLE-SEDDS formulation significantly reduced malondialdehyde (MDA) levels in all examined tissues, demonstrating their protective role against lipid peroxidation. No significant differences were observed between the two vehicle groups. However, in the kidney, the PLE-SEDDS formulation exhibited a markedly superior effect in reducing MDA levels compared to the extract, further highlighting its enhanced efficacy (Figure 4g–i).

In the liver, MDA levels decreased by 28.9% with the extract treatment, whereas the PLE-SEDDS formulation achieved a greater reduction of 44.7%. Similarly, in the kidney, MDA levels declined by 42.4% with the extract and by 59.8% with PLE-SEDDS, reinforcing its superior antioxidant potential. In the pancreas, although MDA reductions were comparable between the extract and PLE-SEDDS groups, the formulation still exhibited a slightly higher decrease, with reductions of 62.8% and 67.6%, respectively.

### 3.9. Serum Lipid Profile

This analysis showed that diabetic animals treated with the *P. ligularis* extract and PLE-SEDDS significantly decreased their plasma concentrations of total triglycerides, cholesterol, and LDL-C, while HDL-C levels increased. Specifically, the total triglyceride levels in the vehicle and SEDDS vehicle groups were 225.5 ± 13.4 mg/dL and 231.6 ± 4.3606 mg/dL, respectively, while the optimized extract and its corresponding formulation reported values of 83.9 ± 8.5 mg/dL and 78.2 ± 11.6 mg/dL. In the cholesterol parameter, the standardized extract and PLE-SEDDS decreased levels by around 27.9% and 31.0% compared to their respective vehicles. LDL levels in the vehicle groups were around 180 mg/dL, while the extract and its formulation presented values of 116 and 109 mg/dL, being statistically different. Finally, HDL-C levels increased slightly by 17% in animals treated with the extract and 26% in those administered with PLE-SEDDS (Table 1).

## 4. Discussion

Research on phyto-drug delivery systems for diabetes remains scarce compared to other pathologies such as cancer, infections, neurodegenerative diseases, and cardiovascular diseases [19,20]. Moreover, conventional formulations are often insufficient to promote adequate release, bioavailability, and pharmacokinetics of phyto-drugs, primarily due to the poor water solubility and rapid metabolism of many natural compounds in the gastrointestinal tract [5]. These challenges are particularly relevant for antidiabetic substances, such as flavonoids and other secondary plant metabolites, whose low bioavailability limits their clinical effectiveness. Advanced drug delivery systems, such as SEDDSs, have emerged as a promising strategy to overcome these limitations and maximize the therapeutic potential of drugs [21], especially bioactive ingredients derived from natural compounds or plant extracts [5,22]. This approach is supported by the results from our study, which demonstrate significant modulation of blood glucose levels, attenuation of the weight loss process, and improved oral glucose tolerance outcomes in diabetic animals treated with PLE-SEDDS during the chronic study period (Figure 1 and Figure 2). When glucose metabolism is improved, it prevents the degradation of proteins and lipids reservoirs, and therefore attenuates diabetes-induced weight loss [23], a result worth highlighting for the PLE-SEDDS group which showed a nearly negligible weight loss (0.2%).

In addition to the known mechanisms of action of *O*-glycosylated flavonoids in *P. ligularis*, which act as insulin secretagogues and enhance insulin sensitivity [9,10], the PLE-SEDDS formulation demonstrated superior glucose control (Figure 2). This enhanced effect can be attributed to the unique mechanisms by which SEDDSs improve the absorption efficiency of active compounds. One pathway is bypassing first-pass metabolism through lymphatic transport. Upon forming a micro- or nanoemulsion in the gastrointestinal tract, the triglycerides in the lipophilic phase undergo lipolysis, yielding diglycerides, monoglycerides, and emulsified fatty acids. In the presence of bile acids, these components form mixed micelles, which are absorbed by enterocyte-formed chylomicrons. Large chylomicrons bypass blood capillaries and enter lymphatic vessels, enabling drugs in SEDDSs to avoid first-pass metabolism and reach systemic circulation via the internal jugular and left subclavian veins [24].

Another critical factor that determines the ability of SEDDSs to permeate mucus barriers is the composition of the formulation, along with the resulting droplet size and zeta potential of the nano-emulsion [7,25]. The PLE-SEDDS formulation comprises castor oil, Cremophor^®^ EL, propylene glycol, and PDMSHEPMS [14]. The latter is a silicone copolymer (poly[dimethylsiloxane-co-[3-(2-(2-hydroxyethoxy)ethoxy)propyl]methylsiloxane]) incorporated to enhance mucus permeation of glycosylated flavonoids through a slippery mechanism, as the polymer provides a muco-inert coating, facilitating the droplets’ passage through the mucus layer [26]. Furthermore, Cremophor EL, a nonionic surfactant that inhibits the P-glycoprotein (P-gp)-mediated efflux system, enhances the intestinal absorption of poorly absorbable drugs that would otherwise be actively secreted by P-gp from the cells back into the intestinal lumen [27,28].

Zeta potential is related to the importance of nanoparticle charge in influencing the oral absorption of encapsulated drugs. The negatively charged nature of the mucus gel, attributed to sulfonic and sialic acid substructures, serves as a barrier for nanoparticles by impeding the diffusion of positively charged particles due to electrostatic interactions. In contrast, negatively charged nanoparticles, such as those in the PLE-SEDDS (−10.92 ± 0.42 mV), are less hindered by these interactions, enabling more efficient permeation through the mucus layer and potentially improving drug bioavailability [14,29,30].

Droplet size also influences the interaction of the nano-emulsion with the mucus layer. Previous research has shown that droplet sizes typically below 50 nm enhance penetration through absorption sites via the transcellular pathway [31]. This is further supported by the findings of Friedl et al., where SNEDDSs (self-nano-emulsifying drug delivery systems) droplets around 12 nm exhibited significantly higher diffusion potential (70%) through mucus membranes compared to larger droplets (450 nm), which achieved only 8% diffusion [32]. This can be attributed to smaller droplets providing a larger interfacial surface area for interaction with the cell membrane, which is strongly associated with enhanced drug release and absorption efficiency [33,34]. The PLE-SEDDS formulation, with a droplet size of 45.93 ± 1.02 nm [14], falls within an optimal size range that likely facilitates efficient mucus penetration of the PLE, maximizing the therapeutic potential of the encapsulated flavonoids.

Additionally, the Log D values for PLE-SEDDS in pH 1.2 and pH 6.8 release media (1.73 ± 0.06 and 2.71 ± 0.11, respectively) suggest minimal flavonoid release in acidic conditions, highlighting the formulation’s ability to safeguard these compounds from acid-induced degradation and ensuring higher absorption availability [14].

The enhanced permeation, in conjunction with the protective properties of the formulation, likely accounts for the superior antihyperglycemic effect of PLE-SEDDS depicted in Figure 2, compared to the standardized extract (PLE). Interestingly, while both the PLE and PLE-SEDDS groups exhibited notable reductions in blood glucose levels compared to vehicle controls, the PLE-SEDDS formulation demonstrated a glycemic response comparable to, and in some parameters even more favorable than, the metformin-treated group. This comparison underscores the potential of PLE-SEDDS as a viable alternative for glycemic control, warranting further exploration in future studies.

Considering that type 2 diabetes mellitus (T2DM) is a chronic metabolic disorder characterized by hyperglycemia, hyperlipidemia, and inflammation—among the most common features contributing to diabetic complications [35]—some authors emphasize the importance of focusing on the broader “antidiabetic” activity of plant-derived treatments rather than solely their “hypoglycemic” effects, given the wider clinical implications [36]. In line with this perspective, this study additionally focused on analyzing the effect of the PLE-SEDDS formulation on the antioxidant and hypolipidemic properties previously demonstrated by aqueous extracts of *P. ligularis* leaves [12].

All of the above diabetic conditions contribute to tissue damage, particularly affecting the size and function of pancreatic β-cells [37]. The protective mechanisms observed in the histological analyses of the PLE and PLE-SEDDS groups are reflected in the well-preserved pancreatic, kidney, and liver tissues. Specifically, the preservation of endocrine components associated with pancreatic islets, as shown in Figure 3e-f, highlights the maintenance of functional pancreatic β-cells responsible for insulin secretion. This pancreatic tissue protection likely contributes to the improved glycemic control, lipid profile, and oxidative stress markers observed in diabetic mice across both treatment groups. Furthermore, this finding aligns with the HOMA-IR values, which indicate that animals treated with PLE and PLE-SEDDS decreased insulin resistance. Remarkably, the HOMA-IR index for the PLE-SEDDS group was lower than the one observed in the metformin-treated control group, highlighting its effectiveness. The enhanced performance of the PLE-SEDDS can be attributed to its ability to improve the bioavailability of bioactive flavonoids, such as isoquercetin and astragalin, which are known to protect pancreatic β-cells and improve insulin sensitivity [38,39,40].

The increased activity of antioxidant enzymes (SOD and CAT) and the reduction in MDA levels in the evaluated organs support the protective role of the treatments against oxidative stress. Specifically, CAT protects pancreatic beta cells by neutralizing H_2_O_2_ [41]. The enhanced hepatic and renal SOD activity and significantly increased CAT activity in all tissues for the PLE-SEDDS suggest that the active components of the extract exhibit higher antioxidant activity, particularly in H_2_O_2_ metabolism. The results emphasize the enhanced efficacy of PLE-SEDDS compared to PLE, suggesting that the improved bioavailability of flavonoids leads to a more robust antioxidant response, making the self-emulsifying systems a more effective alternative than standardized unformulated extracts.

Conversely, elevated MDA levels are indicative of peroxidative damage, often associated with diabetic complications and diminished antioxidant defense mechanisms [42]. The ability of the PLE-SEDDS formulation to inhibit MDA levels more effectively than the standardized extract highlights its superior protective effect against lipid peroxidation. Similar results have been reported for isoquercetin, one of the primary glycosylated flavonoids associated with the antidiabetic effects of *P. ligularis*. The flavonoid prevented the decline of antioxidant enzymes in an STZ-induced diabetic rat model, likely by preserving pancreatic tissue and function, as well as regulating Nrf2 pathway-associated genes, including SOD and CAT. Isoquercetin additionally alleviated hyperlipidemia and inflammation [43].

In T2DM, insulin resistance impairs the regulation of lipid metabolism, leading to metabolic disturbances such as increased lipolysis, which elevates circulating levels of free fatty acids [44]. Additionally, there are alterations in the lipid profile characterized by elevated triglyceride (TG) and LDL-C levels, alongside reduced HDL-C levels [45,46]. The absence of insulin resistance in animals treated with the standardized extract and PLE-SEDDS likely contributed to the observed improvements in lipid profiles (Table 1). These include reductions in TG, total cholesterol, and LDL-C levels, along with an increase in HDL-C, compared to diabetic animals treated with vehicles. Notably, the PLE-SEDDS formulation demonstrated a greater hypolipidemic effect than the PLE.

In recent years, nanomedicine-based phyto-drug delivery systems have gained considerable attention in diabetes treatment, with self-emulsifying drug delivery systems (SEDDSs) emerging as a promising strategy to enhance the bioavailability of bioactive compounds, such as flavonoids. However, research on SEDDSs for delivering plant-derived extracts with antidiabetic properties remains limited. Our findings provide strong evidence supporting SEDDSs as an effective platform for incorporating biologically active molecules from natural product extracts. By improving glycemic control, reducing oxidative stress, and addressing lipid imbalances, the PLE-SEDDS formulation offers a novel and promising therapeutic approach for managing the complex complications of diabetes.

## 5. Conclusions

Finally, this study reinforces the importance of advancing drug delivery systems, such as the PLE-SEDDS formulation, for improving the therapeutic potential of natural extracts in diabetes management. Notably, the PLE-SEDDS formulation demonstrated superior efficacy in modulating glycemic control, oxidative stress parameters, and lipid profile, which can be attributed to the enhanced systemic concentrations of flavonoids achieved through its self-emulsifying matrix. Additionally, the preserved structural integrity of the pancreas, kidneys, and liver, in the groups treated with the extract and PLE-SEDDS formulation, highlights their potential safety and compatibility in treated rodents. However, further pharmacokinetic studies in animals and humans are essential to confirm the bioavailability enhancement of the bioactive compounds in *P. ligularis* mediated by the SEDDS formulation. Such studies would provide critical insights into the correlation between improved bioavailability and the observed efficacy, solidifying PLE-SEDDS as a potential, novel strategy for diabetic treatment.

## Figures and Tables

**Table 1:** Effects of PLE-SEDDS on the lipid profile in diabetic mice induced by the model of repeated doses of streptozotocin and high-fat diet.

Treatment	Triglycerides mg/dL	Cholesterol mg/dL	LDL-C mg/dL	HDL-C mg/dL
Normoglycemic	96.87 ± 2.23 ****	140.28 ± 15.35 ****	88.82 ± 25.78 ****	37.843 ± 1.93 ***
Vehicle	225.51 ± 13.35	252.07 ± 5.48	174.66 ± 17.53	28.923 ±0.08
SEDDS vehicle	231.59 ± 4.36	245.39 ± 12.52	179.09 ± 18.89	28.433 ± 0.37
Metformin 250 mg/kg	169.61 ± 3.62 ****	159.76 ± 4.81 ****	98.44 ± 13.63 ***	36.727 ± 4.82 **
*P. ligularis* extract 250 mg/kg	83.88 ± 8.47 ****	181.93 ± 16.33 ****	116.97 ± 6.26 **	33.860 ± 1.33 *
PLE-SEDDS	78.20 ± 11.67 ^++++^	159.73 ± 13.96 ^++++^	109.35 ± 9.00 ^+++^	35.913 ± 0.38 ^++^

LD-C: Low-density lipoprotein, HDL-C: high-density lipoprotein. Data expressed as mean ± SEM, *n* = 6 animals per group. One-way ANOVA followed by Dunnet’s post-test; * *p* < 0.05; ** *p* < 0.01; *** *p* < 0.001; and **** *p* < 0.0001 compared to vehicle group. One-way ANOVA followed by Dunnet’s post-test; ^++^ *p* < 0.01; ^+++^ *p* < 0.001; and ^++++^ *p* < 0.0001 compared to SEDDS vehicle group.

## Data Availability

The original data and findings presented in this study are included within the article. For additional information or inquiries, please contact the corresponding authors.

## Abbreviations

The following abbreviations are used in this manuscript:

PLE-SEDDS	Self-emulsifying drug delivery system of *Passiflora ligularis*
SEDDS	Self-emulsifying drug delivery system
BGLs	Blood glucose levels
PLE	*P. ligularis* extract
OGTT	Oral glucose tolerance test
SOD	Superoxide dismutase
CAT	Catalase
MDA	Malondialdehyde
MAPKs	Mitogen-activated protein kinases
MEK/ERK	Mitogen-activated protein kinase/extracellular-signal-regulated kinase
GLUT-4	Glucose transporter protein type-4
T2DM	Type 2 diabetes mellitus
ELISA	Enzyme-linked immunosorbent assay
STZ	Streptozotocin
HOMA-IR	Homeostasis model assessment–insulin resistance index
TGs	Triglycerides
HDL-C	High-density lipoprotein cholesterol
LDL-C	Low-density lipoprotein cholesterol
SEM	Standard error of the mean
SD	Standard deviation
ANOVA	Analysis of variance
PDMSHEPMS	Poly[dimethylsiloxane-co-[3-(2-(2-hydroxyethoxy)ethoxy)propyl]methylsiloxane]
ER	Endoplasmic reticulum
GPx	Glutathione peroxidase
TXN	Thioredoxin
